# Development and Characterization of *Magnoliae Flos* Essential-Oil-Loaded Nanoemulsion: A Spatiotemporal Nose-to-Brain Delivery Enhancer for Solution and Gel-Based Pharmaceutical Formulations

**DOI:** 10.3390/pharmaceutics17121535

**Published:** 2025-11-28

**Authors:** Shiyu Zong, Miao Wang, Xinyu Ma, Yunlong Cheng, Ye Li, Hong Zhang, Chunliu Wang

**Affiliations:** 1 Institute of Traditional Chinese Medicine, Shaanxi Academy of Traditional Chinese Medicine, Xi’an 710003, China; zongsy114@163.com (S.Z.); yunlong532@126.com (Y.C.); liyelsj@163.com (Y.L.); zhanghong919919@163.com (H.Z.); 2 Key Laboratory of TCM Drug Delivery, Shaanxi Academy of Traditional Chinese Medicine, Xi’an 710003, China; 3 Pharmacy Department, Xi’an Daxing Hospital, Xi’an 710082, China; wm2835918788@163.com; 4 School of Pharmacy, Shaanxi University of Chinese Medicine, Xianyang 712046, China; maxinyu819@163.com

**Keywords:** *Magnoliae Flos* (Xinyi), nose-to-brain, essential oil, nanoemulsion, Coumarin 6, sulfo-Cy7 carboxylic acid, supramolecular hydrogel

## Abstract

**Objective:** To develop a stable nanoemulsion loaded with *Magnoliae Flos* essential oil (MEO-NE) and evaluated its potential as an enhancer for nose-to-brain delivery in both solution and gel formulations. **Methods:** The MEO-NE was prepared using a low-energy emulsification method, with the formulation optimized via single-factor experiments and Box–Behnken design-response surface methodology. The optimized MEO-NE was characterized for particle size, PDI, morphology, and nasal mucosal irritation. Ex vivo histological imaging in rats was performed using hydrophilic sulfo-cyanine7 carboxylic acid and lipophilic coumarin 6 as fluorescent probes to assess distribution and retention in the trigeminal nerve and brain tissues. **Results:** The optimized MEO-NE exhibited a small particle size (27.96 ± 0.94 nm), low PDI (0.089 ± 0.013), spherical morphology, a stable O/W structure, and no irritation to the nasal mucosa. Ex vivo imaging revealed that MEO-NE significantly enhanced the distribution and retention of both hydrophilic and lipophilic probes in the trigeminal nerve and brain tissues. Moreover, the gel formulation of MEO-NE demonstrated superior brain-targeting efficiency over the solution within 6 h. **Conclusions:** MEO-NE served as an effective enhancer for nose-to-brain delivery, improving brain uptake of both hydrophilic and lipophilic drugs, and provided an experimental basis for utilizing herbal essential oils in CNS-targeted delivery systems.

## 1. Introduction

Intranasal administration, a non-invasive approach, has garnered significant attention for its unique ability to bypass the blood–brain barrier and facilitate direct drug delivery to the brain [1,2]. This concept intriguingly aligns with the traditional Chinese medicine (TCM) theory that “the nasal orifice connects to the brain” implying that the nasal cavity serves not only as a gateway for pathogenic invasion but also as a potential pathway for drug delivery to the central nervous system. With a long history of clinical application, TCM nasal therapy often utilizes aromatic and orifice-opening herbs to guide therapeutic effects upward to the brain, aiming to restore consciousness, repel turbidity, and unblock orifices [3]. Contemporary studies have further indicated that volatile oils or essential oils extracted from medicinal herbs exhibit potential in modulating central nervous system functions, providing a scientific basis for aromatherapy in managing brain disorders [4,5]. Consequently, several aromatic or orifice-opening TCM materials have been explored for nose-to-brain delivery, representing a convergence of traditional knowledge and modern pharmaceutical technologies [6,7].

*Magnoliae Flos* (Xinyi), the dried flower bud of *Magnolia biondii* Pamp., *M. denudata* Desr., or *M. sprengeri* Pamp., is a key TCM for unblocking nasal orifices, traditionally used for conditions like nasal congestion and headache [8,9]. Its volatile oil is considered a primary active component [10,11]. Research suggests that *Magnoliae Flos* volatile oil (MEO) possesses favorable volatility and nasal mucosal permeability, facilitating rapid distribution and absorption along the nose-brain pathway, thereby indicating its potential as a natural penetration enhancer [12]. However, the specific mechanisms underlying its promotion of nose-to-brain transport remain incompletely elucidated, limiting its further application. Furthermore, similar to many volatile oils, MEO faces challenges such as poor stability and potential irritation to the nasal mucosa, which hinder its direct pharmaceutical application [13,14]. Therefore, developing suitable carrier systems to enhance its stability and reduce irritation, while systematically investigating the biological mechanisms behind its traditional “orifice-unblocking” effect, holds significant theoretical value and clinical translation potential.

Nanoemulsions, as thermodynamically stable colloidal delivery systems, have been widely utilized to improve the loading capacity and in vivo performance of volatile oils, owing to their small particle size (typically 1–100 nm), high stability, and good biocompatibility [15,16,17]. Regarding intranasal formulation options, solution-based systems (e.g., sprays, drops) offer advantages of ease of use and uniform distribution [18], whereas gel-based systems can prolong mucosal residence time and provide sustained release, thereby enhancing drug exposure [19,20]. Notably, supramolecular gels with stimuli-responsive properties (e.g., thermosensitivity) show particular promise in nasal drug delivery due to their good bio-adaptability and controlled release capabilities [21,22].

Based on this rationale, this study aims to encapsulate MEO into a nanoemulsion system (MEO-NE) prepared via a low-energy emulsification method, and to systematically characterize its physicochemical properties. Furthermore, the MEO-NE will be incorporated into both solution and gel formulations to comprehensively evaluate its influence on the nose-to-brain delivery of hydrophilic and lipophilic model drugs, thereby elucidating its penetration enhancement mechanism from a spatiotemporal distribution perspective. This work is expected to provide a modern scientific interpretation of the traditional “orifice-unblocking” effect of *Magnoliae Flos* and lay a foundation for developing efficient and safe nose-to-brain co-delivery systems.

## 2. Materials and Methods

### 2.1. Materials

*Magnoliae Flos* slices were purchased from Shaanxi Xingshengde Pharmaceutical Co., Ltd. (Xi’an, China). Coumarin 6 (C6) and sulfo-cyanine7 carboxylic acid (Cy7, water-soluble) fluorescent probes were employed for ex vivo imaging system (IVIS) studies and utilized as model lipophilic and hydrophilic drugs, respectively. These compounds were commercially sourced from Shanghai Maokang Bio-Technology Co., Ltd. (Shanghai, China). Accessories of oleic acid, ethyl oleate, Cremophor RH40, and Cremophor EL35 were obtained from BASF (China) Co., Ltd. (Shanghai, China). Polyethylene glycol 400 (PEG400) and propylene glycol were purchased from Jiangxi Ipsen Pharmaceutical Co., Ltd. (Ji’an, China). The supramolecular hydrogels (Gel) were prepared in our lab according to the previously established method. Other reagents were of analytical grade or better.

Male Sprague Dawley (SD) rats (280 ± 20 g) were purchased from Chengdu Dossy Experimental Animals Co., Ltd. (Chengdu, China). The protocol approval number is SCXK (Sichuan) 2020-0030. All rats were housed in standard cages with wood shavings under controlled environmental conditions (22 ± 1 °C, RH 60 ± 5%) with a 12 h light/dark cycle. They were provided with a standard diet and ad libitum access to water throughout the study. All animal experiments were approved by the Animal Ethics Committee of Shaanxi Academy of Traditional Chinese Medicine and were performed in accordance with the Guide for the Care and Use of Laboratory Animals such as animal welfare, animal protection and ethical standards.

### 2.2. Extraction and Chemical Characterization of MEO

The MEO was extracted by steam distillation. Specifically, 100 g of *Magnoliae Flos* slices were crushed into fine powder and placed in a 2000 mL distillation flask, then 1000 mL of deionized water was added and mixed. Boiling stones were added to prevent bumping. After 30 min of static immersion, the mixture was subjected to controlled heating (maintained at gentle boiling) for 2 h using a standard essential oil extraction apparatus. Following extraction, the system was cooled for 60 min before collecting the essential oil layer. Three independent replicates were performed. The obtained oil was dehydrated with anhydrous sodium sulfate and precisely weighed to determine the extraction yield (*ER*, %), calculated as:

$$ER\%=M_{V}/M_{M}\times 100\%$$

where *M_V_* represents the mass of extracted essential oil (g) and *M_M_* denotes the initial mass of *Magnoliae Flos* slices (g).

The chemical composition of MEO was analyzed using Shimadzu GC-MS-QP2010 Plus (Shimadzu Corporation, Tokyo, Japan) under the following analytical conditions:

Gas chromatography conditions: Separation was achieved on an Agilent DB-5 capillary column (30 m × 0.25 mm × 0.25 μm). The temperature program was initiated at 50 °C, increased to 131 °C at a rate of 3 °C/min, then to 181 °C at 2 °C/min, and finally to 241 °C at 10 °C/min, with a 1 min hold at the final temperature. The injector temperature was set to 260 °C, and helium carrier gas was delivered at a constant flow rate of 1 mL/min. A split ratio of 5:1 and a solvent delay time of 3.5 min were applied.

Mass spectrometry conditions: Electron ionization mode was operated at 70 eV. The ion source, interface, and quadrupole temperatures were maintained at 230 °C, 270 °C, and 150 °C, respectively. Mass spectra were acquired in full-scan mode over a range of *m*/*z* 30–550.

### 2.3. Preparation and Optimization of MEO-NE

The MEO-NE was prepared using a spontaneous emulsification method. Briefly, the oil phase (containing MEO as a constituent of the oil component), emulsifier, and co-emulsifier were blended at a predetermined mass ratio within a 25 mL beaker. The mixture was homogenized under continuous magnetic stirring at 350 rpm for 15 min to achieve a uniform pre-concentrate. Subsequently, deionized water was titrated dropwise into the system under gentle agitation until a homogeneous dispersion exhibiting characteristic bluish opalescence (Tyndall effect) was observed, confirming the formation of the nanoemulsion. Notably, the MEO was directly incorporated into the oil phase without additional solvent modification to preserve its native physicochemical properties.

The systematic optimization of the MEO-NE formulation was performed through a two-stage strategy. Initially, single-factor experiments were conducted to screen emulsifiers (Cremophor RH40 and EL35), co-emulsifiers (propylene glycol and PEG 400), and other oil phases (oleic acid and ethyl oleate) except MEO, followed by preliminary optimization of mass ratios: mixed oil phase compositions (9:1 to 1:9), emulsifier-to-co-emulsifier ratios (Km = 3:1 to 1:2), and mixed oil phase-to-mixed emulsifier ratios (9:1 to 1:9). Subsequently, a Box–Behnken design (BBD) based on response surface methodology (RSM) was implemented using Design-Expert 8.0.5.0 software, with particle size as the dependent variable (Y). Independent variables included (A) mixed oil phase-to-mixed emulsifier ratio, (B) mixed oil phase composition, and (C) Km value. Quadratic polynomial models derived from BBD were validated via ANOVA, lack-of-fit tests, and *r* analysis, while 3D surfaces elucidated factor interactions. Triplicate parallel validation experiments were conducted to verify the optimal formulation predicted by the models, ensuring the reliability and accuracy of the experimental results.

### 2.4. Characterization of MEO-NE

The appearance of MEO-NE was visually inspected under natural light. For particle size, polydispersity index (PDI), and zeta potential analysis, the MEO-NE was diluted 100-fold with deionized water and measured using a Nano-ZS Zen 3600 Zetasizer (Malvern Instruments Co., Ltd., Malvern, UK). The morphology of MEO-NE was determined using transmission electron microscope (TEM) (Hitachi H-7500, Hitachi Ltd., Tokyo, Japan). The sample was deposited onto a carbon-coated copper grid. After staining with 2% phosphotungstic acid solution, excess stain was removed using filter paper, followed by air-drying at room temperature prior to TEM imaging.

The nanoemulsion type was determined through a dual-method approach [23,24]: (1) Two aliquots of the same MEO-NE batch were separately stained with Sudan III (lipophilic dye) and methylene blue (hydrophilic dye). The emulsion type was classified as oil-in-water (O/W) if methylene blue exhibited faster diffusion than Sudan III, or water-in-oil (W/O) if the reverse pattern was observed; (2) Phase stability was assessed by mixing MEO-NE with deionized water-retention of homogeneous appearance indicated O/W type, while phase separation suggested W/O type. Additionally, the pH value was determined at room temperature using an FE20 digital pH meter (Mettler-Toledo Instruments Co., Ltd., Shanghai, China) with triplicate measurements.

### 2.5. Quantification of MEO Content

A standard stock solution of MEO (2.0 mg/mL) was prepared by accurately transferring 20 mg of MEO into a 10 mL volumetric flask, followed by dilution with methanol to the mark and ultrasonication for homogenization. Serial dilutions of the stock solution with methanol yielded standard working solutions with concentrations of 20, 30, 40, 50, 60, 70, and 80 μg/mL. The absorbance values (*A*) were measured at 233 nm using a Hitachi U-2910 UV-Vis spectrophotometer (Hitachi Ltd., Tokyo, Japan) with methanol as the blank. A linear regression analysis was performed by plotting the absorbance (*A*) against the concentration of MEO (*C*) to establish the calibration relationship.

For sample analysis, the prepared MEO-NE formulation was accurately weighed (*M_T_*) and diluted with methanol to a defined volume (*V*). Absorbance measurements were conducted at 233 nm in triplicate. The concentration of MEO (*C_M_*) was determined using the established calibration equation, and the drug loading capacity (DL, %) was calculated according to the following formula:

$$DL\%=C_{M}\times V/M_{T}\times 100\%$$

### 2.6. Stability Study

The stability of MEO-NE was systematically evaluated under multiple conditions. For centrifugal stability assessment, samples were subjected to low-speed (4000 rpm) and high-speed (12,000 rpm) centrifugation for 20 min, followed by visual inspection for phase separation, creaming, or coalescence, with particle size and PDI quantitatively analyzed. Thermal stability studies involved incubating MEO-NE at 4 °C, 25 °C, and 37 °C for 24 h, with physicochemical changes visually recorded and colloidal parameters (size, zeta potential, PDI) measured. Long-term stability was assessed by storing MEO-NE under ambient conditions for 2 months, with samples collected at 0, 1, and 2 months to evaluate morphological integrity and quantify colloidal properties.

### 2.7. Nasal Mucosal Irritation Test

To assess local irritation, SD rats were randomly assigned to a saline control or an MEO-NE group (*n* = 3). Following a single unilateral intranasal administration of 100 μL, the nasal septal mucosa was collected after 24 h for H&E staining.

### 2.8. Effect of MEO-NE on Intranasal Brain Delivery of Solution-Formulated Drug

SD rats (*n* = 120) were randomly allocated into four groups (*n* = 30/group): Cy7 solution, MEO-NE@Cy7, C6 suspension, and MEO-NE@C6. Each group underwent sampling at five time points (10 min, 30 min, 2 h, 3 h, 6 h; *n* = 6 per time point). Probe formulations were prepared as follows: Cy7 was dissolved in physiological saline to prepare a 2 mg/mL solution, while C6 was vortex-ultrasonically dispersed (200 W, 5 min) in saline (5 mg/mL suspension). For the MEO-NE groups, either Cy7 or C6 was incorporated into MEO-NE through continuous magnetic stirring at 350 rpm for 5 min, resulting in homogeneous drug-loaded systems with identical concentrations. Under 2% pentobarbital anesthesia, rats received unilateral intranasal administration (100 μL) in supine position using a micropipette. At predetermined time points, trigeminal nerves (ophthalmic and maxillary branches) and whole brains (including olfactory bulb, cerebellum, and brainstem) were dissected for ex vivo imaging. Fluorescence imaging was performed using an IVIS Spectrum imaging system (PerkinElmer, Shelton, CT, USA) in Epi-Illumination mode, with excitation/emission wavelengths of 747 nm/774 nm for Cy7 and 444 nm/505 nm for C6. Fluorescence intensity, quantified as average radiant efficiency (ARE), was measured via region-of-interest (ROI) analysis [25].

### 2.9. Effect of MEO-NE on Intranasal Brain Delivery of Gel-Formulated Drugs

The gel formulations were prepared in our laboratory through host-guest interactions between sodium carboxymethyl cellulose (CMC-Na) and carboxymethyl-β-cyclodextrin (CS-β-CD). Briefly, the base gel was formulated by hydrating 90 mg of sodium carboxymethyl cellulose (CMC-Na) in 2.5 mL of water overnight. Separately, 10 mg of carboxymethyl-β-cyclodextrin (CS-β-CD) was dissolved in 1.25 mL of water. These solutions were combined and homogenized at 350 rpm until a transparent, viscous gel formed. For the MEO-NE-loaded gel (MEO-NE@Gel), the 1.25 mL water used to dissolve CS-β-CD was replaced with 1.25 mL of MEO-NE, followed by identical mixing with the hydrated CMC-Na solution. Fluorescent probe-loaded gels (Cy7@Gel and C6@Gel) were subsequently prepared by directly dissolving Cy7 or C6 into the plain gel base, resulting in homogeneous mixtures with final probe concentrations of 2 mg/mL and 5 mg/mL, respectively. Finally, dual-loaded gels (MEO-NE@Cy7@Gel and MEO-NE@C6@Gel) were formulated by dissolving Cy7 or C6 directly into the pre-formed MEO-NE@Gel, achieving homogeneous systems containing both MEO-NE and the fluorescent probe at the specified concentrations (2 mg/mL Cy7, 5 mg/mL C6).

SD rats (*n* = 120) were randomly assigned to four groups (*n* = 30/group): Cy7@Gel, MEO-NE@Cy7@Gel, C6@Gel, and MEO-NE@C6@Gel. Each group was further divided into subgroups (*n* = 6 per subgroup) corresponding to five sampling time points: 10 min, 30 min, 2 h, 3 h, and 6 h. Following anesthesia, rats were placed in a supine position and administered a unilateral intranasal dose of 100 µL of the respective gel formulation. At each designated time point, rats were sacrificed, and the trigeminal nerve and brain tissues were carefully dissected. The distribution of the fluorescent probes (Cy7 or C6) within the dissected trigeminal nerve and brain regions was then visualized and analyzed using an IVIS imaging system. ARE of tissues was determined via the ROI quantification method.

### 2.10. Statistical Analysis

Statistical analyses were carried out using GraphPad Prism 8.2 software. The significance of differences between groups were analyzed using one-way ANOVA with the Student-Newman-Keuls test. The results were presented as means ± standard deviation, and *p* value < 0.05 was considered statistically significant.

## 3. Results

### 3.1. Chemical Composition of MEO

The MEO was extracted by steam distillation. The extracted MEO was a pale yellow, clear, and transparent oily liquid, with an average extraction yield calculated to be 2.3%. The obtained MEO was analyzed by GC-MS, and the resulting total ion chromatogram (Figure S1) was used for compound identification by searching against the NIST Version 17.L mass spectral library. A total of 115 compounds were identified, among which 28 compounds exhibited relative peak areas exceeding 1%. The major components included camphor, cadinol, camphene, terpineol, isopiperitenol, cadinene, 1,8-Cineole, etc [26]. Detailed compositional information was provided in Table 1.

### 3.2. Preparation of MEO-NE

**Figure 1:**
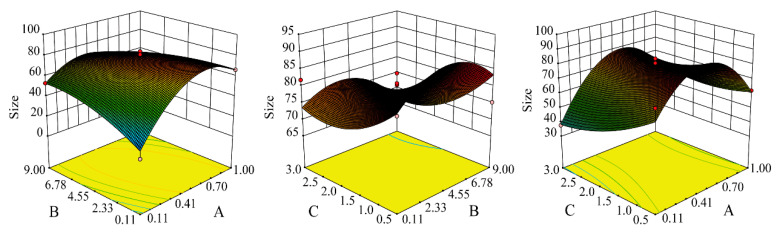
Three-dimensional response surface plots depicting the interactive effects of factors on the particle size of MEO-NE.

Single-factor experiments showed that MEO-NE with small particle size and low PDI was achieved using a mixture of ethyl oleate and MEO as the oil phase, Cremophor EL35 as the emulsifier, and PEG400 as the co-emulsifier, with a MEO-to-ethyl oleate ratio of 1:1, a Km of 2:1, and an oil-to-emulsifier ratio of 2:3. A BBD with 17 runs was then used for further optimization. The resulting nanoemulsion particle sizes ranged from 18.21 nm to 83.81 nm. The experimental data were fitted to a second-order polynomial regression model, yielding the equation as *Y* = 78.93 + 4.2*A* − 2.37*B* − 6.22*C* − 14.32*AB* + 7.24*AC* − 2.05*BC* − 26.58*A*^2^ − 7.56*B*^2^ + 5.08*C*^2^ (*r =* 0.9598). ANOVA confirmed the model’s significance (*p* < 0.001), and the lack-of-fit test was not significant (*p* > 0.05), indicating the model can reliably predict particle size. Interaction analysis (Figure 1) and model predictions led to the optimal formulation: MEO:Ethyl oleate:Cremophor EL35:PEG400:H_2_O = 1:1:3:2:10.

Validation experiments conducted in triplicate using the optimal formulation showed minimal deviation between measured and predicted particle sizes. Excellent reproducibility was also observed across the three batches (RSD < 3%). These results confirmed that the optimized MEO-NE preparation process is robust and reproducible.

### 3.3. Characterization of MEO-NE

**Figure 2:**
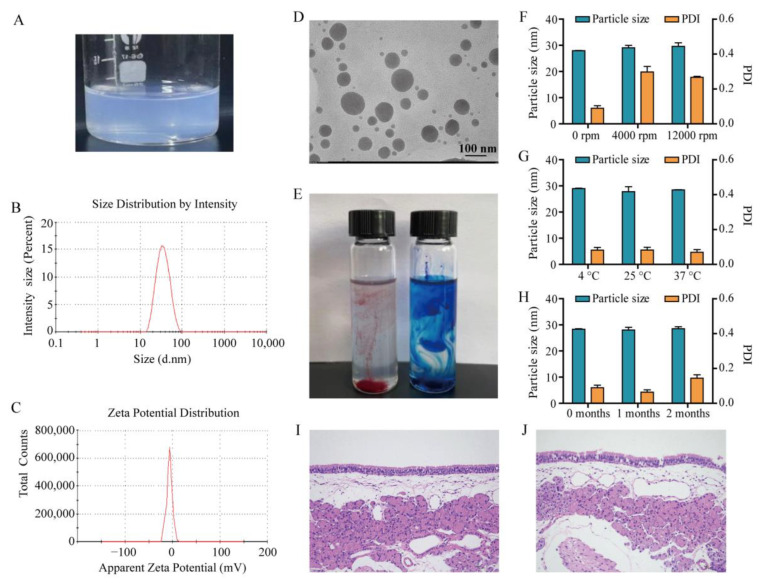
Characterization and nasal irritation assessment of MEO-NE. (**A**) Appearance. (**B**) Size distribution. (**C**) Zeta potential. (**D**) TEM image. (**E**) Oil/water type confirmation (Sudan III, red; Methylene Blue, blue). (**F**–**H**) Stability under centrifugation, heating, and long-term storage. (**I**,**J**) Histopathological images of nasal mucosa from rats treated with (**I**) saline and (**J**) MEO-NE (HE stain, ×200).

The prepared MEO-NE was a clear, transparent solution with faint blue opalescence (Figure 2A). The particle size, PDI and zeta-potential of MEO-NE were 27.96 ± 0.94 nm, 0.089 ± 0.013, −2.82 ± 0.7 mV, respectively (Figure 2B,C). The TEM images further substantiated the monodisperse spherical architecture of nano-droplets (diameter < 100 nm) with negligible aggregation (Figure 2D), which aligned well with the dynamic light scattering measurements. For nanoemulsion type identification, MEO-NE displayed rapid diffusion in the aqueous methylene blue dye system (Figure 2E) and maintained structural integrity upon aqueous dilution without phase separation, collectively confirming its O/W nature. The pH value of the system was measured to be 6.37 ± 0.06.

### 3.4. Determination of MEO Content

Linear regression analysis of absorbance (*A*) versus MEO concentration (*C*) yielded the calibration equation *A* = 0.0042*C* + 0.0047 (*r* = 0.9990). The result demonstrated that MEO exhibited a good linear relationship within the concentration range of 20 to 80 μg/mL. The mean DL% of MEO in MEO-NE was calculated as 5.86% with high precision (RSD = 0.07%).

### 3.5. Stability Evaluation

The stability assessment revealed that MEO-NE maintained excellent physical integrity without exhibiting phase separation, creaming, or coalescence under all tested conditions. Notably, while a moderate increase in PDI was observed under centrifugal stress, the particle size distribution remained remarkably consistent across all evaluation parameters (Figure 2F–H). These findings collectively demonstrate the formulation’s exceptional resistance to centrifugal forces, thermal degradation, and long-term storage challenges. The comprehensive stability profile confirms that the MEO-NE developed in this study possesses excellent overall stability and is well-suited for further application in subsequent research.

### 3.6. Evaluation of Nasal Mucosal Irritation

The administration was well-tolerated across all animals, with no observed adverse reactions such as sneezing, asthma, coughing, or choking. Histopathological examination revealed well-arranged cilia and intact mucosal architecture in both groups. Compared to the control group, the MEO-NE group showed no signs of tissue damage, significant inflammatory cell infiltration, or other notable histopathological alterations (Figure 2I,J). These results indicate that MEO-NE does not induce obvious irritation to the nasal mucosa.

### 3.7. Effect of MEO-NE on the Nasal-to-Brain Distribution of Solution and Gel Formulations

To investigate the effect of MEO-NE on the nasal-to-brain distribution of drugs in solution and gel formulations, rats were euthanized at various time points post-administration. The trigeminal nerves and brain tissues were collected and examined using an IVIS imaging system to detect the fluorescence intensity of the probes Cy7 (representing a hydrophilic drug) and C6 (representing a lipophilic drug).

**Figure 3:**
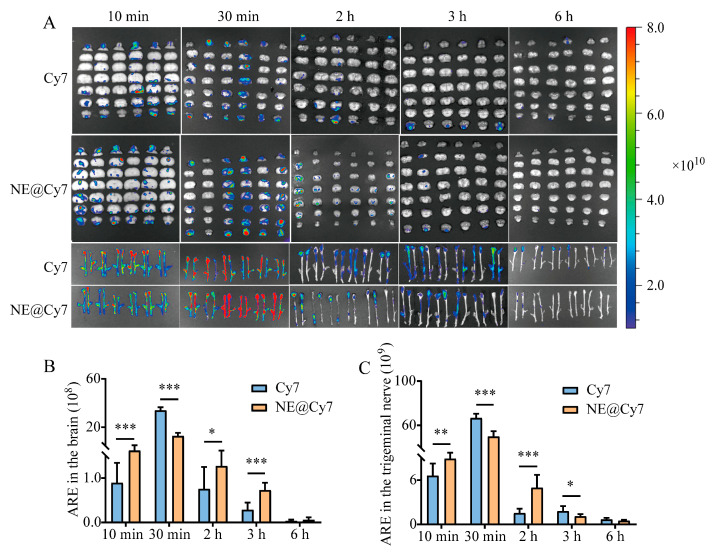
Ex vivo fluorescence imaging (**A**) and quantitative analysis (**B**,**C**) of Cy7 distribution in the brain and trigeminal nerve at various time points after intranasal delivery of Cy7 solution or MEO-NE@Cy7. Abbreviations: ARE, Average Radiant Efficiency [(p/s/cm^2^/sr)/(μW/cm^2^)]. Values are statistically significant at * *p* < 0.05, ** *p* < 0.01, *** p < 0.001 (*n =* 6).

For the solution formulation, the Cy7 results showed that the strongest fluorescence intensity in both the brain and trigeminal nerve was observed at 30 min after administration (Figure 3A). Quantitative analysis revealed that in the brain, ARE values of the NE@Cy7 group were higher than those of the Cy7 group at all time points except 30 min, with significant differences at 10 min, 2 h, and 3 h (*p* < 0.001, *p* < 0.05; Figure 3B). In the trigeminal nerve, the NE@Cy7 group exhibited significantly higher ARE values than the Cy7 group at 10 min and 2 h (*p* < 0.01, *p* < 0.001; Figure 3C).

**Figure 4:**
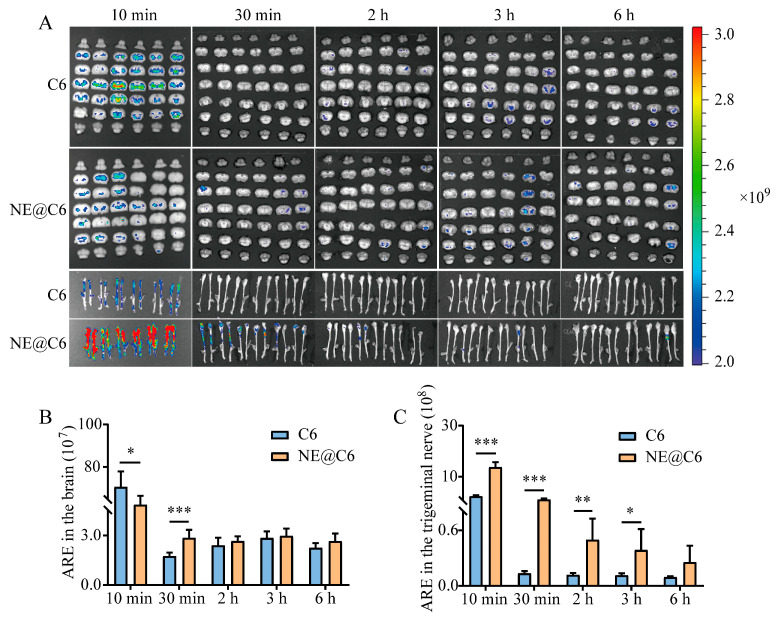
Ex vivo fluorescence imaging (**A**) and quantitative analysis (**B**,**C**) of C6 distribution in the brain and trigeminal nerve at various time points after intranasal delivery of C6 suspension or MEO-NE@Cy7. Abbreviations: ARE, Average Radiant Efficiency [(p/s/cm^2^/sr)/(μW/cm^2^)]. Values are statistically significant at * *p* < 0.05, ** *p* < 0.01, *** p < 0.001 (*n* = 6).

The C6 results indicated that the highest fluorescence intensity in the brain and trigeminal nerve occurred at 10 min after administration (Figure 4A). ARE analysis showed that in the brain, the NE@C6 group had higher ARE values than the C6 group at all time points except 10 min, with a highly significant difference at 30 min (*p* < 0.001; Figure 4B). In the trigeminal nerve, the NE@C6 group demonstrated higher ARE values than the C6 group at all time points, with extremely significant differences at 10 min and 30 min (*p* < 0.001), and significant differences at 2 h and 3 h (*p* < 0.01, *p* < 0.05; Figure 4C).

**Figure 5:**
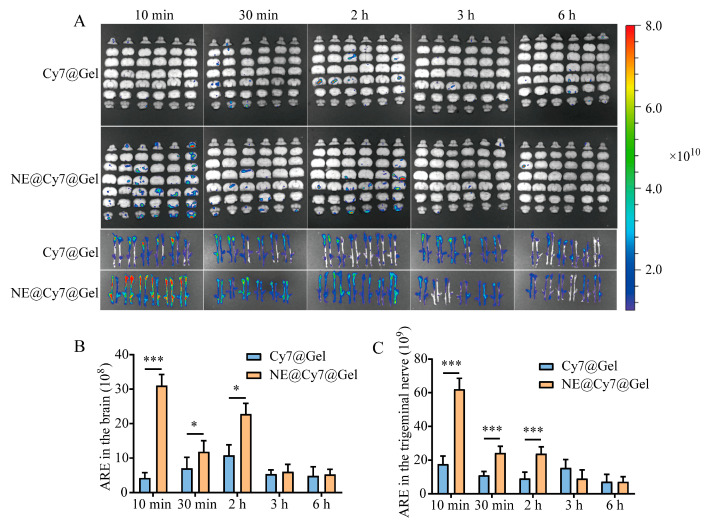
Ex vivo fluorescence imaging (**A**) and quantitative analysis (**B**,**C**) of Cy7 distribution in the brain and trigeminal nerve at various time points after intranasal delivery of Cy7@Gel or MEO-NE@Cy7@Gel. Abbreviations: ARE, Average Radiant Efficiency [(p/s/cm^2^/sr)/(μW/cm^2^)]. Values are statistically significant at * *p* < 0.05, *** p < 0.001 (*n =* 6).

For the gel formulation, IVIS images of Cy7 showed that at 10 min, the fluorescence intensity of the NE@Cy7@Gel group was stronger than that of the Cy7@Gel group in both the brain and trigeminal nerve (Figure 5A). ARE analysis indicated that in the brain, the NE@Cy7@Gel group had higher ARE values at all time points, with a highly significant difference at 10 min (*p* < 0.001), and significant differences at 30 min and 2 h (*p* < 0.05; Figure 5B). In the trigeminal nerve, the NE@Cy7@Gel group showed significantly higher ARE values than the Cy7@Gel group at 10 min, 30 min, and 2 h (*p* < 0.001; Figure 5C).

**Figure 6:**
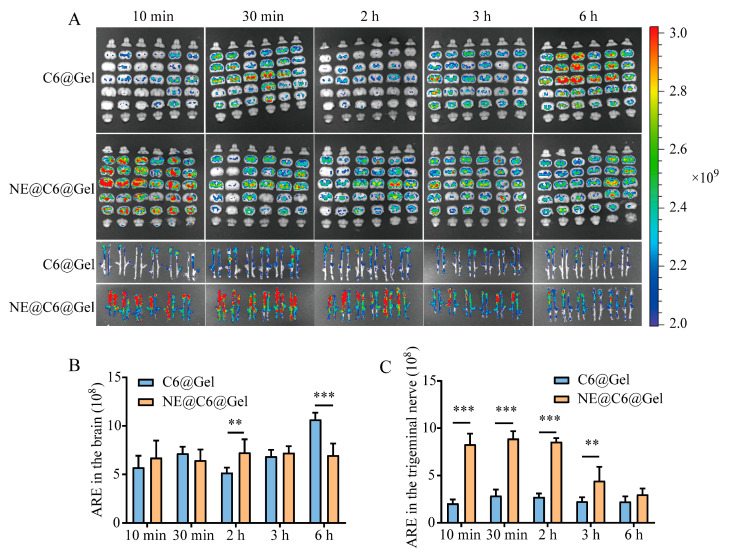
Ex vivo fluorescence imaging (**A**) and quantitative analysis (**B**,**C**) of C6 distribution in the brain and trigeminal nerve at various time points after intranasal delivery of C6@Gel or MEO-NE@C6@Gel. Abbreviations: ARE, Average Radiant Efficiency [(p/s/cm^2^/sr)/(μW/cm^2^)]. Values are statistically significant at ** *p* < 0.01, *** p < 0.001 (*n =* 6).

The IVIS images of C6 are shown in Figure 6A. ARE results demonstrated that in the brain, the NE@C6@Gel group exhibited higher ARE values at 10 min, 2 h, and 3 h, with a significant difference at 2 h (*p* < 0.01; Figure 6B). In the trigeminal nerve, the NE@C6@Gel group showed significantly higher ARE values than the C6@Gel group at all time points except 6 h (*p* < 0.01, *p* < 0.001; Figure 6C).

**Figure 7:**
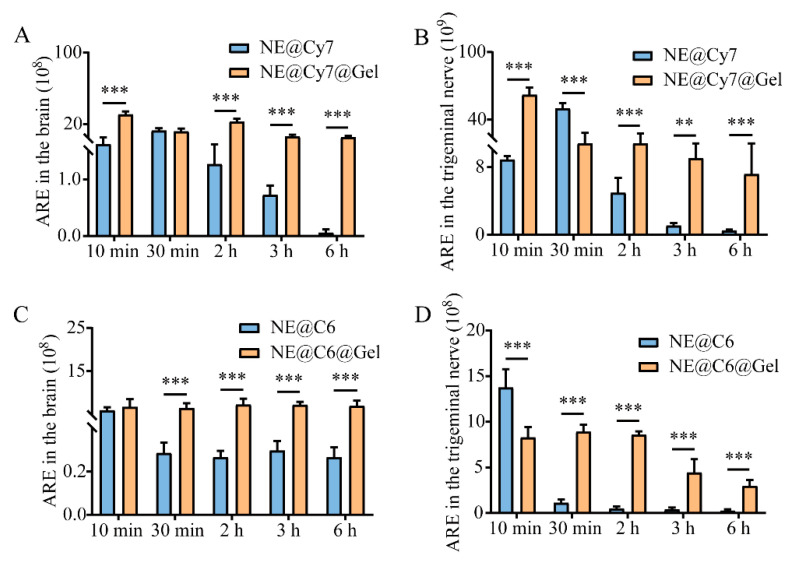
Quantitative analysis of fluorescence intensity in the brain and trigeminal nerve at various time points following administration of solution or gel formulations. (**A**) ARE of Cy7 in the brain; (**B**) ARE of Cy7 in the trigeminal; (**C**) ARE of C6 in the brain; (**D**) ARE of C6 in the trigeminal nerve. Abbreviations: ARE, Average Radiant Efficiency [(p/s/cm^2^/sr)/(μW/cm^2^)]. Values are statistically significant at ** *p* < 0.01, *** p < 0.001 (*n =* 6).

Further comparison of the formulation types revealed that for Cy7, the fluorescence intensity of the NE@Cy7@Gel group was significantly higher than that of the NE@Cy7 group in both the brain and trigeminal nerve at all time points except 30 min (*p* < 0.01, *p* < 0.001; Figure 7A,B). For C6, the fluorescence intensity of the NE@C6@Gel group was significantly higher than that of the NE@C6 group in both regions at all time points except 10 min (*p* < 0.001; Figure 7C,D).

## 4. Discussion

This study successfully developed MEO-NE via a low-energy emulsification method and systematically evaluated its impact as a nose-to-brain delivery enhancer on the distribution of model drugs in both solution and gel formulations. The results demonstrated that MEO-NE significantly increased the distribution and retention time of both the hydrophilic probe Cy7 and the lipophilic probe C6 in rat trigeminal nerve and brain tissues, with the gel formulation exhibiting superior delivery efficiency compared to the solution.

The successful preparation and optimization of the nanoemulsion formed the foundation of this research. The strategy combining single-factor experiments and BBD-RSM was employed to efficiently and precisely determine the optimal formulation [27,28]. This approach not only significantly enhanced the stability of the nanoemulsion but also successfully yielded a system with a small particle size (approximately 28 nm) and a narrow size distribution (PDI < 0.1). These small, uniform nanoemulsion droplets are conducive to promoting interaction with the local mucosa, thereby enhancing drug penetration and absorption [29]. Characterization results confirmed that the prepared MEO-NE was an O/W-type nanoemulsion with uniform spherical morphology, a near-neutral zeta potential, and a pH value compatible with the physiological nasal environment (typically pH 5.5–6.5), providing a solid foundation for intranasal administration [30,31]. Furthermore, MEO-NE exhibited good stability under centrifugation, thermal stress, and long-term storage conditions, indicating its potential for practical application.

For the in vivo distribution evaluation, Cy7 and C6 were selected as model probes for hydrophilic and lipophilic drugs, respectively. Ex vivo fluorescence imaging enabled visual and quantitative comparison of the distribution of different formulations in the trigeminal nerve and brain following intranasal administration. The results indicated that the incorporation of MEO-NE significantly increased the exposure (represented by ARE values) and retention time of both probes in the target tissues, regardless of the formulation type (solution or gel). These findings not only corroborate the traditional efficacy of MEO in “opening orifices with its aromatic property and directing therapeutics upward” but also provide a modern pharmaceutics basis for understanding its mechanism. It is hypothesized that this effect may be related to the volatile oil promoting mucosal penetration, modifying drug partitioning behavior at the nasal mucosa [12], and potentially facilitating pathways associated with the olfactory or trigeminal nerves [32]. Given that MEO itself possesses documented anti-inflammatory and anti-allergic pharmacological activities [33,34], its dual role as both a penetration enhancer and an active component warrants further investigation.

Compared to the solution formulation (MEO-NE), the gel formulation (MEO-NE@Gel) achieved significantly higher delivery efficiency across most time points. We speculate that this improvement stems from a combination of factors. Firstly, the gel matrix significantly extends the nasal residence time of MEO-NE, reducing mucociliary clearance and enabling sustained release for more thorough mucosal interaction [35,36]. Secondly, CS-β-CD, a known absorption enhancer present in the formulation, is expected to further promote drug uptake into neural tissues and subsequent distribution within the brain [37,38]. The combination of nanoemulsions with a gel system thus creates a synergistic platform that enhances the overall effectiveness of brain-targeted delivery [39]. Importantly, the pronounced facilitation of lipophilic drug delivery to the brain and trigeminal nerve highlights the potential of this strategy for lipophilic therapeutics and for treating diseases with foci in the trigeminal nerve.

Nevertheless, this study has certain limitations. Firstly, the specific molecular mechanisms by which MEO-NE enhances nose-to-brain delivery remain to be fully elucidated. Secondly, the study utilized healthy animal models, and delivery efficacy under pathological conditions (e.g., inflammation, barrier impairment) was not investigated. Finally, while the developed MEO-NE showed no signs of nasal irritation in this acute setting, its long-term safety profile warrants further evaluation. To build upon these findings, several directions are proposed for future work. A direct comparative assessment of the nasal irritation potential between pure MEO and MEO-NE would provide definitive evidence for the improved safety of the nanoemulsion system. Furthermore, employing relevant olfactory cell line models could help dissect the cellular mechanisms—such as modulation of tight junctions or enhancement of endocytic uptake—underlying the permeability-enhancing effect of MEO-NE.

## 5. Conclusions

In conclusion, this study has successfully developed a safe, stable, and efficient *Magnoliae Flos* essential oil-loaded nanoemulsion (MEO-NE) system and confirmed its potential as a functional excipient for nose-to-brain delivery, significantly enhancing the distribution and retention of both hydrophilic (sulfo-Cy7 carboxylic acid, Cy7) and lipophilic (Coumarin 6, C6) model drugs in rat trigeminal nerve and brain tissues. Importantly, the composite gel delivery system formed by incorporating MEO-NE into a sodium carboxymethyl cellulose (CMC-Na)/carboxymethyl-β-cyclodextrin (CS-β-CD) gel matrix demonstrated advantages over the simple solution formulation in terms of prolonging nasal residence, controlling release, and promoting brain-targeted delivery. In summary, this research not only provides a modern pharmaceutical basis for the traditional efficacy of *Magnoliae Flos* essential oil (MEO) but also develops a promising nanoemulsion-gel composite delivery system with clinical application potential, offering a new strategy for utilizing herbal volatile oils as natural penetration enhancers in nose-to-brain targeted delivery.

## Figures and Tables

**Table 1:** Components in MEO (accounting for >1%).

No.	Retention Time (min)	Compound	Peak Area	Relative Percentage (%)	CAS Registry No.
1	6.886	β-Ocimene	364,011,868	2.73	13877-91-3
2	7.118	(−)-Camphene	377,367,712	2.83	5794-04-7
3	7.397	6-(4-fluorophenyl)-4,5-diazaspiro[2.4]hept-4-ene	436,562,133	3.27	920338-72-3
4	7.519	(+)-Camphene	341,839,731	2.56	5794-03-6
5	7.92	1,2,3,4,5-Pentamethylcyclopentadiene	160,909,813	1.21	4045-44-7
6	7.988	(3,4-Dimethylphenyl)methanol	188,819,137	1.42	6966-10-5
7	8.135	cis-Chrysanthenyl formate	635,510,838	4.77	241123-18-2
8	8.23	(1R,2R)-1,2-di(prop-1-en-2-yl)cyclobutane	307,782,589	2.31	19465-02-2
9	8.295	1,8-Cineole	230,189,679	1.73	470-82-6
10	8.496	3-Methyl-6-propan-2-ylidenecyclohexene	245,013,511	1.84	586-63-0
11	8.844	(+)-4-Carene	170,050,531	1.28	29050-33-7
12	8.950	L-Fenchone	160,050,083	1.20	7787-20-4
13	9.036	Linalool	397,927,991	2.98	78-70-6
14	9.882	(−)-Camphor	833,156,796	6.25	464-48-2
15	10.020	2,3,3-Trimethylbicyclo(2.2.1)heptan-2-ol	233,568,079	1.75	465-31-6
16	10.260	Terpinen-4-ol	270,655,589	2.03	562-74-3
17	10.469	(−)-α-Terpineol	450,728,955	3.38	10482-56-1
18	11.611	(−)-Bornyl Acetate	182,898,316	1.37	5655-61-8
19	13.563	2-Methylene-4,8,8-trimethyl-4-vinylbicyclo[5.2.0]nonane	297,129,756	2.23	242794-76-9
20	14.180	γ-Muurolene	197,781,281	1.48	30021-74-0
21	14.348	(1S,4aR,8aS)-1-isopropyl-7-methyl-4-methylene-1,2,3,4,4a,5,6,8a-octahydronaphthalene	411,098,533	3.08	6980-46-7
22	14.475	α-Muurolene	284,519,322	2.13	31983-22-9
23	14.744	(+)-δ-Cadinene	487,297,194	3.65	483-76-1
24	15.515	(2E,4S,7E)-4-Isopropyl-1,7-dimethylcyclodeca-2,7-dienol	268,300,097	2.01	198991-79-6
25	16.295	T-Cadinol	516,214,127	3.87	5937-11-1
26	16.461	α-Cadinol	549,827,089	4.12	481-34-5
27	17.043	1-(3,3-dimethylcyclohexylidene)ethaol	679,676,654	5.10	26532-23-0
28	17.194	(−)-cis-Isopiperitenol	1,156,650,161	8.67	96555-02-1

Note: (+) and (−) denote dextrorotatory and levorotatory optical rotation, respectively.

## Data Availability

The original contributions presented in this study are included in the article/Supplementary Material. Further inquiries can be directed to the corresponding author.

## Supplementary Materials

The following supporting information can be downloaded at: https://www.mdpi.com/article/10.3390/pharmaceutics17121535/s1, Figure S1: Total ion current chromatogram of MEO-NE by GC-MS.
