# Supplementary figures and images for: Development and Characterization of Magnoliae Flos Essential-Oil-Loaded Nanoemulsion: A Spatiotemporal Nose-to-Brain Delivery Enhancer for Solution and Gel-Based Pharmaceutical Formulations
Source: Pharmaceutics. 2025 Nov 28;17(12):1535. doi: 10.3390/pharmaceutics17121535 (PMC12736246; doi:10.3390/pharmaceutics17121535)

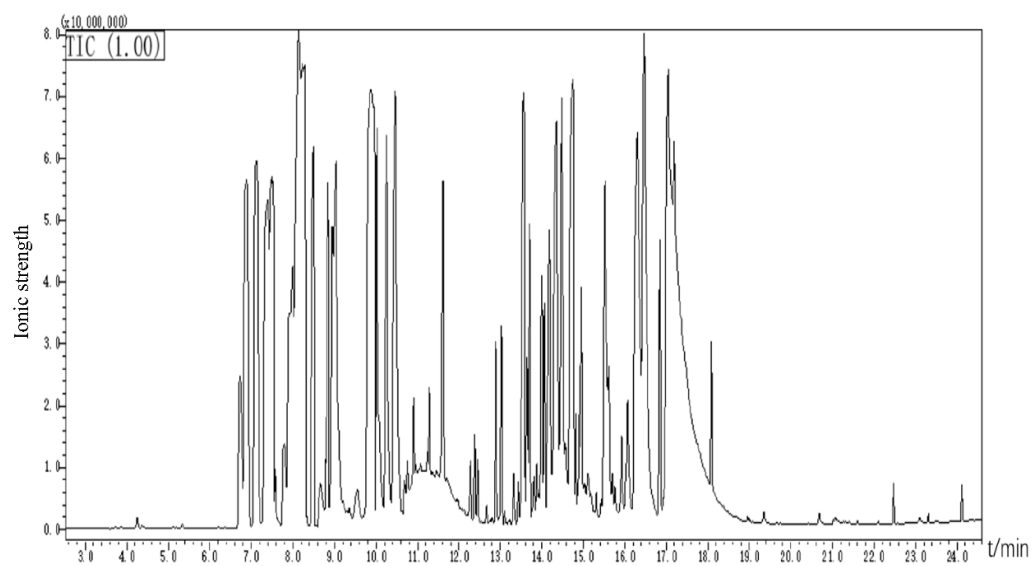

Figure S1. Total ion current chromatogram of MEO-NE by GC-MS.

Supplement: Supplementary file 1 [file pharmaceutics-17-01535-s001.zip › pharmaceutics-3949377-supplementary.pdf]
